# Role of skin autofluorescence in managing renal and cardiac diseases in outpatient dermatology

**DOI:** 10.1111/srt.13211

**Published:** 2022-10-28

**Authors:** Farshid Etaee, Tarek Naguib, Mohamad Goldust, Steven Daveluy, Howard Maibach

**Affiliations:** ^1^ Department of Medicine Yale University New Haven Connecticut USA; ^2^ Department of Medicine Texas Tech Health Sciences Center Amarillo Texas USA; ^3^ Department of Dermatology Yale University New Haven USA; ^4^ Department of Dermatology Wayne State University Detroit Michigan USA; ^5^ Department of Dermatology University of California San Francisco San Francisco California USA

**Keywords:** advanced glycation end products (AGE), end‐stage renal disease, kidney disease, kidney failure, skin autofluorescence (SAF)

## Abstract

**Introduction:**

The accumulation of tissue‐advanced glycation end products in skin results from complex and consecutive reactions and can be measured by skin autofluorescence (SAF) reader devices. This overview discusses studies evaluating the utilization of SAF in screening renal and cardiac disease.

**Materials and methods:**

Literature search was performed using Google Scholar, PubMed, Springer, Ovid, and ScienceDirect.

**Results:**

SAF was an independent predictor of progression of chronic kidney disease (CKD) and was elevated in subjects on hemodialysis and peritoneal dialysis. Furthermore, SAF was significantly associated with cardiovascular events, cardiovascular mortality, and all‐cause mortality in CKD patients. Other studies revealed a correlation between SAF and arterial stiffness, vascular damage, and subclinical atherosclerosis. A vegetarian diet was associated with lower SAF levels, whereas malnutrition was correlated with higher levels and increased mortality.

**Conclusions:**

SAF measurement may be useful in managing renal and cardiac disease. Future studies are needed to clarify the specific role of SAF in the management of CKD and its noninvasive office utilization to identify comorbidities in inflammatory diseases, such as psoriasis.

## 1 INTRODUCTION

Accumulation of tissue‐advanced glycation end products (AGEs) results from complex reactions, including a mixture of hyperglycemia, oxidative/carbonyl stress, or diminished renal clearance of AGE precursors,^1^ and these skin depositions can be identified by skin autofluorescence (SAF).^2^ The association between SAF and cardiovascular, nutritional, and mortality outcomes, among other factors, was assessed in patients with various stages of kidney disease. This extensive review describes the available literature and suggests a possible role in outpatient dermatology, especially in inflammation and following patients with inflammatory skin disease, such as psoriasis.

## 2 MATERIALS AND METHODS

A Google Scholar, PubMed, Springer, Ovid, and ScienceDirect literature search was performed using the following keywords: “glycation end products,” and “SAF,” and “kidney,” “renal,” “cardiac,” “chronic kidney disease (CKD),” “acute kidney disease,” “kidney injury,” “end‐stage renal disease,” “hemodialysis (HD),” “peritoneal dialysis (PD),” “mortality,” “complications,” and “diet.” We attempted to describe all aspects of the correlation between SAF and kidney and cardiovascular diseases (CVDs) and their management. All included publications regarding SAF are summarized in Table 1. In these studies, AGE accumulation was assessed based on the SAF using the autofluorescence reader (AGE reader). The AGE Reader device used to measure SAF was developed at the University Medical Center Groningen, The Netherlands. The AGE Reader is a noninvasive monitoring device that uses ultraviolet light to excite autofluorescence in human skin tissue.

**TABLE 1:** Studies that assessed skin autofluorescence (SAF) and kidney disease

**Manuscript**	**Study design**	**Kidney function**	**Assessment**	**Number of patients, age, and sex**	**Key findings**
Furuya et al.^1^	Prospective study	CKD on HD	Relationship between SAF and CVD in CKD patients	A total of 64 chronic CKD Overall, 73% of the patients were male. The mean age of patients was 60.9 ± 13.2 years	Highest SAF level showed an increased odds ratio for the prevalence of CVD. These findings suggest that SAF levels can be used to detect the prevalence of cardiovascular in HD patients with or without diabetes
Nongnuch et al.^2^	Prospective cohort study	HD patients	The effect of vegetarian diet on SAF in HD patients	A total of 332 adult HD patients. Mean age was 65.2 ± 15.1 years. 64% were male	SAF, a marker of tissue AGE deposition, was reduced in vegetarian HD patients after correction for known confounders, which suggests that a vegetarian diet may reduce exposure to preformed dietary AGE. Dietary manipulation could potentially reduce tissue AGE and SAF as well as CVD risk
Lavielle et al.^3^	Letter	A total of 35 patients admitted for AKI and 35 ESRD	SAF in acute kidney injury	AKI (age: 62 ± 15; 57% women) ESRD (age: 62 ± 10; 54% women)	SAF is lower in AKI than in CKD and relates to the duration of renal failure
Oleniuc et al.^4^	Cross‐sectional study	A total of 157 HD, 102 PD, 32 CKD, and 19 type 2 diabetic patients, without renal failure	Evaluation of AGEs accumulation, using SAF, in CKD and dialysis patients	A total of 310 patients: 157 HD patients (mean age 60 years, 19.1% diabetic), 102 PD patients (mean age 56.3 years, 17.6% diabetic), 32 CKD patients (mean age 68 years, 34.4% diabetic), and 19 type 2 diabetic patients, without renal failure (mean age 59 years)	Tissue AGE values, expressed as SAF, were significantly higher in patients treated with dialysis compared to pre‐dialysis CKD patients. Patients in the control group, although diabetic, but without renal impairment, had the lowest AGE levels. Nevertheless, all these values are significantly higher compared to reference values recorded in healthy subjects, for each age category
Jiang et al.^5^	Cohort study	Maintenance dialysis patients (PD and HD)	Assessing effects of the accumulation of tissue AGEs correlated and glucose exposure dose and cardiovascular morbidity in patients on PD	A total of 2388 maintenance dialysis patients (613 PD and 1775 HD). Mean age was 51.0 ± 15.0 in PD group and 55.1 ± 15.3 HD. Overall, 50.4% were male in PD group and 56.6 in HD group	Compared to HD group, PD patients receiving conventional glucose‐containing dialyzate had significantly higher SAF values in each category of age and dialysis duration, irrespective of the presence or absence of diabetes. In PD patients, SAF values strongly correlated with the duration of PD and glucose exposure dose and independently associated with cardiovascular morbidity. Multivariate analysis revealed that glucose exposure dose and SAF were the strongest risk factors for cardiovascular morbidity in PD patients after adjustment by age, gender, and other classic‐ or uremic‐related risk factors
McIntyre et al.^6^	Cross‐sectional study	A total of 115 established dialysis patients (62 HD and 53 PD)	Evaluation of tissue‐AGE concentration in dialysis patients	A total of 115 established dialysis patients, 62 HD (55% male, age 65.3 ± 15.5), and 53 PD (58% male, age 62.5 ± 13.1)	SAF values in PD and HD patients were similar and strongly correlated with historical PD glucose exposure. SAF correlated with age in both groups but with dialysis vintage only in PD patients
Siriopol et al.^7^	Observational study	A total of 304 dialysis patients (202 on chronic HD and 102 on continuous ambulatory PD)	Comparing tissue‐AGE levels in patients receiving either HD or PD and to study the effect of these products on all‐cause, cardiovascular, or sepsis‐related mortality	A total of 304 dialysis patients with the mean age of 56.7 ± 14.4 and 135 of 304 were male (44.4%)	Accumulation of tissue AGEs in end‐stage kidney disease, as measured by SAF, is not influenced by the dialysis modality. Another important finding of our study is that AGE levels are associated with all‐cause and sepsis‐related mortality in PD patients and only with sepsis‐related mortality in diabetic patients
Bentata et al.^9^	Cross sectional	A total of 444 patients with uncontrolled or complicated type 2 diabetes	Assessment of SAF, renal insufficiency, and retinopathy in patients with type 2 diabetes	A total of 261 patients were men (58.8%). Their mean age was 62.0 ± 10.1 years	In type 2 diabetes, patients with renal insufficiency, the high SAF does not relate to retinopathy
McIntyre et al.^10^	Prospective cohort study	CKD stage 3	SAF and association with renal and cardiovascular risk factors in CKD stage 3	A total of 1707 patients Mean age 72.9 ± 9 years, 61% were female	Increased SAF was independently associated with multiple cardiovascular (CV) and renal risk factors in CKD 3. Long‐term follow‐up will assess the value of SAF as a predictor of CV and renal risk in this population. Age remained an independent determinant of increased SAF
Fraser et al.^11^	Prospective cohort	A total of 1741 people with CKD stage 3	Assessing relationship between SAF and all‐cause mortality in stage 3 CKD	Of 1741 people recruited to the RRID study, 1707 (98%) people had valid measures of skin AF; 34 participants were excluded, because skin AF readings could not be obtained because of dark skin color (*n* = 17) or technical failure (*n* = 17). Overall, mean follow‐up time was 3.66 ± 0.8 years (1317 ± 287 days)	SAF was associated with all‐cause mortality, but this association was lost after adjustment for other known risk factors
Shardlow et al.^12^	Prospective cohort study	A total of 1707 people with relatively mild CKD = stage 3	Association of SAF with cardiovascular events and all‐cause mortality in subjects with CKD stage 3	The mean age was 72.9 ± 9.0 years, 1036 (60.7%) were female	SAF was as an independent risk factor for cardiovascular events and all‐cause mortality in subjects with early CKD. These findings suggest that interventions to reduce AGE accumulation, such as dietary AGE restriction, may reduce cardiovascular risk in CKD
Morohoshi et al.^13^	Cohort study	A total of 199 CKD5 patients (non‐dialysis CKD5, *n* = 100, HD, *n* = 27 and PD, *n* = 72)	Assessment of the relationship among SAF, chronological age, and all‐cause mortality in patients with CKD stage 5	Median age 66 years, 34% females	Biological age calculated by SAF, and chronological age are equally robust predictors of clinical outcomes in CKD; however, both indices are influenced by the inflammatory status
Tanaka et al.^14^	Prospective observational study	A total of 416 pre‐dialysis patients with CKD	Assessing relationship between SAF and the progression of CKD	Mean age of all 416 patients was 64.0 (51.0–74.0) years, 207 (50%) were male	Tissue accumulation of AGE, measured as SAF, was a strong and independent predictor of progression of CKD. SAF may be useful for risk stratification
Tanaka et al.^15^	Cross‐sectional study	A total of 304 pre‐dialysis CKD patients	Assessing relationship among SAF, renal function, and CVDs in pre‐dialysis CKD patients	Median age was 62.0 years (IQR, 49.3–73.0 years), and 51.3% of subjects were male	SAF increased as CKD stage advanced. CVD history showed independent effects on SAF in both the diabetic and nondiabetic groups. Moreover, SAF was higher in patients with CVD than in those without and still showed a significant contribution to CVD in the multivariable logistic regression model that included traditional risk factors for CVD, such as age, smoking, blood pressure, and diabetes
Wang et al.^16^	Prospective cohort study	A total of 245 subjects with at least 2 conventional risk factors for coronary artery disease	Assessing the relationship between SAF and the renal function in subjects at increased risk of coronary artery disease	Mean age of 245 subjects was 64.48 ± 12.24. A total of 148 of 245 were male	SAF could predict renal function decline in a 2‐year follow‐up period in patients at increased risk of CAD. Subgroup analysis showed SAF to be an independent factor for relative renal function decline rate in the settings of age <65 years, male sex, BMI ≧ 25 kg/m^2^, and eGFR ≧ 60 ml/min/1.73 m
Hörner et al.^17^	Prospective observational study	A total of 109 patients on HD and 28 on PD	Assessing the factors associated with change in SAF, a measure of AGE, in subjects receiving dialysis	Enrolled 109 current HD and 28 current PD patients. Mean age of the whole cohort was 65 ± 14 years. A total of 90 (66%) participants were male	SAF increases over time in most subjects receiving dialysis. We also identified that prevalence or development of malnutrition over 1‐year, current smoking, and HD as first dialysis modality were independent predictors of increasing SAF. Of note, dietary AGE intake was not an independent determinant of the 1‐year increase in SAF in this dialysis population
França et al.^18^	Observational, transversal, controlled, single‐center study	HD patients and control	Analyzing AGE measured by SAF levels and its relations with CVD and bone mineral disorder parameters in HD patients	A total of 20 HD patients (HD group) and healthy subjects (control group, *n* = 24)	This study detected a negative correlation of SAF with serum intact parathormone, suggesting a role of AGE on the pathophysiology of bone disease in HD prevalent patients
Tanaka et al.^19^	Cross‐sectional study	Patients on maintenance HD	Associations between SAF, CVD, and other parameters were studied	A total of 128 patients: 59 men and 69 women. Mean age for patients was 65.1 ± 11.6	Increased SAF was related to the presence of CVD in Asian (non‐Caucasian) HD patients, and therefore an autofluorescence reader might have the potential to be a useful assessment of cardiovascular risk
Ramsauer et al.^20^	Crossover, interventional study	A total of 28 patients on chronic HD	Comparing changes in plasma and SAF in low‐flux versus high‐flux HD	A total of 28 patients (20 men and 8 women). The median age of the patients was 67 years (range 37–83 years)	In the dialysis settings used, there was no significant change in skin AF after dialysis, with low‐flux or with high‐flux dialysis. Although only limited reduction in plasma fluorescence was observed, this was more pronounced performing low‐flux dialysis
Nongnuch and Davenport^21^	Prospective observational study	A total of 180 chronic HD patients	Effect of hemodiafiltration, vegetarian diet, and urine volume on AGE products measured by changes in SAF	A total of 180 chronic HD patients, mean age was 66.7 ± 13.9, 65.6% were men	SAF increased with high‐flux HD, whereas SAF did not increase with hemodiafiltration. Residual urine output and vegetarian diet were associated with lower AGE deposition
Ramsauer at al.^22^	A longitudinal interventional study	A total of 24 patients on chronic HD	Skin‐ and plasma autofluorescence in HD with glucose‐free or glucose‐containing dialysate	A total of 24 patients on chronic HD (17 male/7 female). The median age was 70.5 years (range 42–85)	Glucose‐free dialysate may result in a significant reduction of SAF, as a marker of AGE and Amadori products, in contrast to when using glucose‐containing dialysate. The protein‐bound parts of plasma autofluorescence also showed a decrease after 2 weeks. This indicates that it may be possible to hamper or even reverse the deposits of AGE in tissue
Ramsauer et al.^23^	Longitudinal observation study	Patients on chronic HD (15 of those with diabetes)	Checking SAF during winter and summer in dialysis patients	A total of 34 patients on chronic HD were included (24 male). The median age was 68.5 years (range 33–83)	There was at a median 5.6% increase in SAF during the winter period (February–May, *p* = 0.004) and a 10.6% decrease in the SAF during the summer (May–August, *p* < 0.001). HbA1c in the DM rose during the summer (*p* = 0.013). SAF decreased significantly during the summer
Kimura et al.^24^	Prospective observational cohort study	HD patients	SAF and cardiovascular mortality in patients on chronic HD	A total of 128 stable HD patients. The mean age of the subjects was 65.1 ± 11.6 years, 46% were male	SAF, serum albumin, and hsCRP are independent predictors of cardiovascular mortality. However, SAF did not have a significant effect on all‐cause mortality
Gerrits et al.^25^	Cohort study	A total of 105 patients on HD, 23 had diabetes mellitus	Predictive value of SAF on overall and cardiovascular mortality in HD patients	Mean age was 65 years, 68% were male	SAF was an independent predictor of overall mortality in HD patients, but it had no predictive value for cardiovascular mortality
Meerwaldt et al.^26^	Prospective study	HD patients and control	Association of SAF and mortality in HD patients	A total of 109 HD patients. Reference data were obtained from a group of 43 nonsmoking, age‐matched control subjects too. Mean age was 57 ± 16 in HD patient, and 53 ± 16 in control group. Overall, 62% were male in HD, and 47% in control group	SAF is a strong and independent predictor of mortality in ESRD. This supports a role for AGE as a contributor to mortality and CVD and warrants interventions specifically aimed at AGE accumulation
Nongnuch et al.^27^	Observational cross‐sectional study	Adult HD patients	Assessment of the relationship of SAF and mortality in high‐flux HD and HD patients	A total of 332 adult HD patients. Mean patient age was 65.7 ± 15.1 years, 64.2% male, 41.9% diabetic	Accumulation of AGE, measured by SAF, was independently associated with higher risk of death in HD patients. Cardiovascular mortality was the most common cause of death, and although there were more cardiovascular deaths in the higher SAF group, this was not significantly different
Arsov et al.^28^	Prospective study	HD patients	Association of SAF and release of heart‐type fatty acid binding protein in plasma with mortality in HD patients	A total of 169 HD patients. Mean age was 56 years (range 23–82), and 61% were male	Age, hypertension, 1‐year DAF, hsCRP, intercellular adhesion molecule 1, and heart‐type fatty acid binding protein were independent predictors of overall mortality. Hypertension, 1‐year increase in SAF, hsCRP, and heart‐type fatty acid binding protein were also independent predictors of cardiovascular mortality
Mácsai et al.^29^	Cross‐sectional study	A total of 198 PD patients	Assessing factors influencing SAF of patients with PD	A total of 198 patients were on PD (128 were on traditional glucose‐based solutions and 70 patients were partially switched to icodextrin‐based PD)	In addition to age and diabetes, the recent switch to icodextrin‐based PD solution is also a factor associated with increased AGE exposure
Vongsanim et al.^30^	Observational study	A total of 150 consecutive PD patients	Comparison SAF in PD patients using standard versus biocompatible glucose containing peritoneal dialysates	A total of 150 patients; 86 (57.3%) male, median age 62 (53–71) years	Although SAF was lower in PD patients prescribed biocompatible dual chamber dialysates, on multivariable testing, these dialysates were not independently associated with SAF
Mácsai et al.^31^	Prospective observational study	Patients on PD	Assessing relationship of SAF and mortality in patients on PD	A total of 126 patients (mean age 66.2 years, 58% men, diabetes ratio 75/126) had anamnestic CVD (coronary heart disease, cerebrovascular disease, peripheral arterial disease)	Among PD patients, SAF values over 3.61 arbitrary units seem to be a predictor of mortality. The relationship among peritoneal glucose exposure, CVD, and diabetes suggests its suitability to characterize systemic cumulative glucose load in this patient population
Hartog et al.^32^	Prospective study	A total of 285 transplant recipients	Assessing the accumulation of AGEs, measured as SAF, in renal disease	A total of 285 transplant recipients (mean age, 52 years), 32 dialysis patients (mean age, 56 years), and 231 normal control subjects (mean age, 51 years)	Kidney transplantation does not fully correct increased AGE levels found in dialysis patients. The increased AGE levels in kidney transplant recipients cannot be explained by the differences in renal function alone
Crowley et al.^33^	Prospective cohort	A total of 66 patients after a kidney transplant, compared with 1707 patients with CKD stage 3 and 115 patients on dialysis (53 HD and 62 PD)	Evaluation of tissue AGE deposition after kidney transplantation	Transplant (54.2% male, age 53.3 ± 12.4) CKD (52.3% male, age 58.9 ± 9.8) HD (67.9% male, age 66.2 ± 13.5) PD (76.9% male, age 59.7 ± 15.7)	Mean SAF in transplant recipients was significantly lower than in patients on HD and PD but was no different from CKD stage 3. Their data showed a drop in SAF over a meantime of 16 months after transplantation
Hartog et al.^34^	Cross‐sectional study	A total of 285 consecutive renal transplant recipients	Evaluation of the relationship between risk factors for chronic transplant dysfunction and CVD and SAF	A total of 285 consecutive renal transplant recipients (57% male, aged 50 ± 12 years)	SAF was associated with several risk factors for CVD and chronic renal transplant dysfunction
Hartog et al.^35^	Prospective observational study	A total of 302 renal transplant recipients	Assessing relationship of SAF and graft loss in renal transplant recipients	A total of 302 outpatients (age 50 ± 12 years, 45% women)	High SAF values are strongly and independently associated with the development of graft loss in kidney transplant recipients. SAF might be a useful method to estimate the risk for graft loss after kidney transplantation
Calviño et al.^36^	Observational cross‐sectional study	A total of 189 stable renal transplant patients	Assessing relationship between SAF and cardiovascular risk in renal transplant patients	Overall, 61% men, aged 56 ± 13.0 years	In multivariable analysis, age, gender, steroid use, serum phosphate, and handgrip strength remained independently associated with SAF
Makulska et al.^37^	Cross‐sectional multicenter study	Children with CKD and control children. CKD consists of (1) PD, (2) HD, (3) 2–4 stage CKD on conservative treatment	SAF and cardiovascular risk in children with CKD	A total of 76 children with CKD. A total of 26 age‐matched healthy children served as controls	SAF independently predicts cardiovascular and renal risk in diabetes, as well as in CKD
Ueno et al.^38^	Cohort study	A total of 120 Japanese patients with end‐stage renal disease (ESRD) and 110 age‐ and sex‐matched control subjects	Assessing relationship between arterial stiffness in patients with ESRD and SAF	ESRD (73.3% male, age 58.1 ± 9.3) Control (64.5% male, age 57.1 ± 10.5)	A significantly higher pulse wave velocity (PWV), a noninvasive measure of arterial stiffness, and SAF values were noted compared to control
Strozecki et al.^39^	Cross sectional	A total of 24 patients with CKD and diabetic nephropathy, 36 patients with CKD and without diabetes, and 19 controls	Compared SAF and arterial stiffness in patients with diabetic nephropathy and patients with CKD without diabetes	Controls (*n* = 19, 58% male, age 46 ± 13) Nondiabetic CKD (*n* = 36, 58% male, age 51 ± 15) Diabetic CKD (*n* = 19, 58% male, age 46 ± 13)	Accumulation of AGE and arterial stiffness are increased in patients with CKD, particularly in those with diabetic nephropathy; however, the results are not sufficient to confirm the causal role of AGE accumulation in arterial stiffening in CKD
Mukai et al.^40^	Cohort study	Clinically stable patients with CKD stage 5	Relationship of SAF, arterial stiffness, and FRS, and clinical outcome in CKD patients	A total of 261 clinically stable patients with CKD stage 5, including 130 non‐dialyzed and 131 dialyzed treated by PD (*n* = 93) or HD (*n* = 38). Mean age was 56 (29–75), 66% were males	In patients with CKD stage 5, SAF and aortic stiffness associated with mortality, independently of FRS. After adjusting for additional confounders including inflammation, aortic stiffness remained as an independent predictor of outcome. Because the contribution of SAF and aortic stiffness compared with FRS in ROC curve analysis was relatively modest, this underlines the importance of traditional CVD risk factors in CKD
Mac‐Way et al.^41^	Cross‐sectional study	A total of 43 PD were matched to 43 HD based on age, gender, diabetes, and dialysis vintage	Comparison of AGEs, aortic stiffness, and wave reflection in PD versus HD	A total of 43 PD were matched to 43 HD based on age, gender, diabetes, and dialysis vintage. Mean age was 62 ± 13 in PD group and 63 ± 14 on HD group. Overall, 58% male in both groups	SAF and aortic stiffness were higher in PD after adjustments for imbalances in baseline characteristics. Independent of dialysis modality, there was a positive association between SAF, aortic stiffness, and enhanced wave reflection
Makulska et al.^42^	Cohort study	Children with CKD: HD, PD, and patients treated conservatively, and control	SAF and vascular damage in children and adolescents with CKD	A total of 76 children with CKD, of whom 20 children were on HD, 20 were on PD and 36 were treated conservatively A control group of 26 healthy subjects was also included in the study	Results reveal that AGE accumulated in the children with CKD and was related to early vascular changes and a number of biochemical vascular risk markers. SAF measurement, as a noninvasive method, may be useful for the identification of clinical risk factors of vascular disease in CKD children
Wang et al.^43^	Prospective cohort study	A total of 290 patients with stage 3–5 CKD	Assessing relationship between SAF with vascular calcification in CKD patients	The mean age for 290 patients was 60 ± 10. A total of 162 (52%) were male	Tissue AGE, as reflected by SAF, showed a significant novel association with vascular calcification in CKD. These data suggest that increased tissue AGE may contribute to vascular calcification in CKD and diabetes mellitus
Fonseca et al.^44^	Observational and cross‐sectional pilot study	A total of 27 patients with stage 5 CKD on PD	Assessing relationship between AGE accumulation and muscle degeneration and vascular calcification in PD patients	A total of 27 patients aged 48 ± 16 years Overall, 52% were female	Revealed associations between AGE accumulation and lower muscle stiffness/density. Associations that linked muscle degeneration parameters with vascular calcification were observed
Sánchez et al.^45^	Case‐control study	A total of 87 patients with mild‐to‐moderate stages of CKD (glomerular filtration rate from 89 to 30 ml/min/1.73 m^2^) and 87 nondiabetic non‐CKD subjects	Assessing the relationship between AGE and subclinical atherosclerosis at the early stages of CKD	A total of 87 patients with CKD: Age: 58.1 ± 10.6 Women, *n* (%): 33 (37.9) Overall, 87 non‐diabetic non‐CKD subjects: Age: 56.5 ± 8.8 Women, *n* (%): 33 (37.9)	SAF was elevated in patients with mild‐to‐moderate CKD in comparison with control subjects. This finding is related to the presence of subclinical atheromatous disease and appears to be independently associated with the GFR. Therefore, SAF is an easy, fast, and noninvasive method that may help to accurately evaluate real cardiovascular risk in the early stages of CKD
Hartog et al.^46^	Cross‐sectional study	A total of 43 dialysis patients	Assessing the relationship between SAF and diastolic function in dialysis patients	Age 58 ± 15 years, of whom 65% were male	Tissue AGEs measured as skin‐AF, but not plasma AGE levels, were related to diastolic function in dialysis patients
Hörner et al.^47^	Single‐center cross‐sectional study	HD patients	Relationship of SAF with nutritional factors in subjects receiving HD	A total of 120 participants on HD. Median age was 65 (interquartile range 54–75) years. Overall, 76% of the participants were male	Elevated SAF was not positively associated with dietary AGE intake in subjects on HD but was associated with several markers of malnutrition, suggesting that malnutrition is a more important determinant of increased SAF than AGE ingestion in this population. Diabetes, smoking, serum albumin, handgrip strength, protein intake, and dialysis vintage were independent predictors of increased SAF
Hörner et al.^48^	Observational nonrandomized study	A total of 28 patients on dialysis, 27 patients on HD and one on PD	Assessing the impact of dietetic intervention on SAF and nutritional status in subjects receiving dialysis	A total of 28 patients on dialysis, age (years): 65 (IQR 56–74) Male, *n* (%): 14 (50)	Dietetic support was associated with stable SAF levels in this proof‐of‐principal study despite an increase in dietary AGE intake, suggesting that interventions to improve nutrition may be important in preventing the rise in SAF observed in malnourished dialysis populations
Hörner et al.^49^	Prospective cohort study	Patients on dialysis	Assessing effects of SAF and malnutrition on mortality in subjects receiving dialysis	A total of 120 HD and 31 PD. The mean (SD) age of the whole cohort was 64 (14) years. A total of 64% of the population were male	Higher SAF and malnutrition were both significant and independent predictors of increased mortality in subjects receiving dialysis. Several markers of malnutrition, such as serum albumin, handgrip strength, and energy, protein, and fat intake, were important determinants of higher mortality, and that SAF was significantly increased among those dialysis patients who died and were malnourished
Arsov et al.^50^	Prospective study	A total of 156 HD patients	Assessing influence of body mass index on the accumulation of AGEs in HD patients	Mean age of the 156 HD patients was 56 years (range 19–84). Among them, 97 (62%) were male	Calorie, protein, and AGE intakes hardly influence the 1‐year increase of skin AF in HD patients. BMI of HD patients of around 24 kg/m^2^ resulted in a lower 1‐year increase of skin AF
Tangwonglert et al.^51^	Observational study	Patients dialyzing with an arteriovenous fistula	Comparison of SAF in the fistula and non‐fistula arms of patients treated by HD	The total of 267 HD patients, 161 males (60.3%), mean age 63.6 ± 15.0 years	Arteriovenous fistula alters blood flow in the arm, and we found that SAF measurements were lower in the arm with arteriovenous fistula. We suggest that SAF measurements are made in the non‐AVF arm
Chaudhri et al.^52^	Observational study	A total of 139 adult patients attending routine outpatient dialysis	Assessing the pitfalls in the measurement of SAF to determine tissue AGE content in HD patients	A total of 139 adult HD outpatients, mean age 66.5 ± 15.2 years; 62.6% male	SAF measurements could not be readily obtained from a substantial proportion of patients with deeply pigmented skin, and even some Caucasoids. In addition, we observed differences in SAF measured in the right and left forearms in those patients dialyzing with left‐sided arteriovenous fistulae, with higher values recorded from the right arm. In addition, SAF decreased more in the fistula arm for those dialyzing with left‐sided arteriovenous fistulae post‐dialysis. This suggests that measurements are made pre‐dialysis in the non‐fistula arm

Abbreviations: AGE, advanced glycation end products; CKD, chronic kidney disease; CVD, cardiovascular disease; eGFR, estimated glomerular filtration rate; ESRD, end‐stage renal disease; FRS, Framingham risk score; HD, hemodialysis; PD, peritoneal dialysis.

### 2.1 SAF correlation with various types of kidney conditions

Lavielle et al. found SAF to be elevated in subjects with ESRD compared with those with AKI, in whom SAF was still higher than in normal subjects.^3^

In HD subjects, SAF values were higher than in those on PD, whereas all subjects on dialysis displayed higher SAF levels in comparison with those with CKD and those with diabetes without renal impairment.^4^ Although SAF levels in subjects receiving PD with conventional glucose‐containing dialysate were significantly higher than in subjects on HD,^5^ the difference was not consistent between the two modalities in all studies.^6^, ^7^ However, as some studies did not report dialysis clearance and the effects of various dialysates on SAF levels, this issue cannot be evaluated.

It was accepted previously that the development of retinopathy preceded nephropathy development.^8^ Of interest, however, higher SAF levels did not correlate with retinopathy in subjects with type 2 diabetes and CKD. This argues against the value of using SAF to predict diabetic retinopathy as the high values of SAF were also reported in subjects with CKD without diabetes.^9^

Further, a 10‐year prospective large cohort study revealed that CKD stage 3 was associated with significantly higher SAF values in males, diabetics, those with a history of CVDs, anemia, smoking, and proteinuria. Age, diabetes, and estimated glomerular filtration rate (eGFR) were the strongest independent determinants of SAF in CKD. Therefore, SAF may be used as an effective single, noninvasive measurement to predict renal function outcome and CVD events.^10^

Although SAF was not independently correlated with all‐cause mortality in CKD stage 3 in one prospective study with a median of 3.6 years follow‐up,^11^ a similar size cohort of subjects with CKD stage 3 revealed that higher SAF value correlated with a 12% increased risk of events (heart attack, heart failure, or stroke) and a 16% higher risk of all‐cause mortality with a follow‐up of 5–6 years.^12^

Interestingly, biological age, which is assessed by a person's physical and mental functions and influenced by various factors such as diet, exercise, and stress, determined by SAF, was correlated with chronological age in subjects with CKD stage 5; all‐cause mortality was absolutely correlated with biological age determined by SAF and chronological age when adjusted for sex, nutritional status, diabetes, and CVD. Both types of age are evenly strong predictors of outcomes in CKD, although both indices are significantly influenced by the inflammatory status, such as IL‐6 and hsCRP.^13^

In pre‐dialysis CKD population, SAF was an independent predictor of progression of CKD, despite adjustment for age, sex, diabetes, smoking history, and eGFR,^14^ whereas a larger cross‐sectional study on pre‐dialysis CKD patients revealed SAF to negatively correlate with eGFR.^15^ Age, male sex, SAF, smoking, and eGFR were significantly related to CVD risk.^15^, ^16^

While HD, malnutrition, and current smoking were independent factors for SAF elevation, Horner et al. demonstrated that HD influences plasma AGE values, but not SAF scores suggesting that it may take months on HD therapy to influence SAF levels.^17^ This may reflect the time needed for AGE skin deposition. On the other hand, the initial SAF level was an independent risk factor for CVD in subjects on HD with or without diabetes and can be used to predict the prevalence of CVD in this population,^1^ but a smaller study on 20 HD subjects did not reproduce these findings, possibly due to small size.^18^ Interestingly, carotid intima‐media thickness, hsCRP, and SAF were identified as significant risk factors for CVD in subjects on HD from Japan.^19^

Ramsauer et al. demonstrated low‐flux versus high‐flux HD patients had no significant difference in SAF after dialysis.^20^ Moreover, a single‐session high‐flux HD did not change SAF values,^18^ which rose over time in subjects on high‐flux HD. There was also no significant difference in SAF in those solely managed by hemodiafiltration.^21^

A noteworthy decline in SAF levels was reported in 24 HD subjects during standard therapy with glucose‐containing dialysate after switching to a glucose‐free dialysate for 2 weeks, indicating both the potential implication of glucose in the accumulation of AGE and the potential clearance of AGE by short‐term HD.^22^ SAF measurements in a 1‐year follow‐up of subjects on HD uncovered an unanticipated meaningful reduction of SAF during the summer season, not correlated to specific variations in dialysis efficiency. Sun exposure was inversely correlated with CVD and all‐cause mortality in a dose‐dependent fashion; however, the potential advantageous impact of sun exposure mandates further study.^23^

Several cohorts with 2–6‐year follow‐up periods on HD revealed that higher SAF consistently and independently correlated with enhanced mortality, with CVD mortality being the most prevalent.^24^, ^25^, ^26^, ^27^ The increase in SAF value over a year (∆AF) in HD subjects was seven‐to‐ninefold higher than in healthy controls suggesting a 1‐year increase of SAF as an independent predictor of cardiovascular and overall mortality in HD population.^28^

In PD subjects, SAF associates with CKD duration,^4^, ^5^ dialysis vintage (length of time on dialysis), anuria,^4^ and higher glucose exposure.^6^ Furthermore, a cross‐sectional study compared 70 patients on icodextrin‐based PD and 128 on traditional glucose‐based solutions showed subjects’ age, current diabetes status, and icodextrin use to significantly enhance SAF levels.^29^ Comparison of SAF in standard versus biocompatible glucose‐containing peritoneal dialysates revealed that although SAF was lower in PD subjects on biocompatible dialysates, a multivariable analysis showed these dialysates were not independently related to SAF.^30^

SAF levels also independently correlated with CVD in PD subjects and along with glucose exposure dose, SAF was the strongest risk factor for CVD morbidity after adjustment for age, gender, and other uremic‐related risk factors.^5^ Those PD subjects with CVD history turned out to have higher values of SAF compared to those without CVD history.^4^ In PD subjects, high SAF levels predicted all‐cause mortality with and without CVD^31^ and correlated with sepsis‐related mortality.^7^

SAF values were lower in transplant recipients than in dialysis subjects, albeit still higher than controls, suggesting that kidney transplantation only partially corrects elevated SAF levels observed in dialysis subjects.^32^ Interestingly, a large study showed SAF in transplant recipients was significantly lower than in HD and PD subjects but was no different from CKD stage 3 subjects with a reduction in SAF noted over a mean period of 16 months after transplantation,^33^ likely suggesting that many transplant recipients have reduced creatinine clearance. SAF correlates with transplant recipients’ age, systolic blood pressure, hsCRP, duration of pretransplant dialysis, and smoking but correlates negatively with plasma vitamin C levels, creatinine clearance at baseline, and change in creatinine clearance after transplant. No significant impact of immunosuppressive therapy or the application of ACE inhibitors was observed.^34^ SAF remained an independent predictor of graft loss and mortality as separate endpoints in kidney transplant recipients in an average follow‐up of 5.2 years. Accordingly, SAF may be useful to evaluate the risk for graft loss after kidney transplantation.^35^

In stable renal transplant recipients, SAF levels are elevated and correlate with CVD risk. Besides age and male sex, phosphate overload, steroid use, and nutritional status are important factors that correlate with AGE accumulation. Subclinical vascular atheromatosis (allograft resistivity index and ankle‐brachial index) and the REGICOR scale that predicts 10‐year mortality risk from the cardiac disease were also associated with elevated SAF levels.^36^

In children with CKD, SAF was also found to be significantly elevated compared with healthy controls, and the HD group displayed the highest levels. Moreover, in the CKD group, a correlation was noted between SAF and left ventricular mass index, dialysis duration, and blood pressure. This reinforces the role of SAF in the pathogenesis of cardiac myocyte remodeling and links it with blood pressure levels.^37^

### 2.2 Vascular effects

In Japanese subjects with ESRD, a significantly higher pulse wave velocity (PWV), a noninvasive measure of arterial stiffness, and SAF values were noted compared to control,^38^ whereas the same trend was identified in CKD subjects with diabetic nephropathy suggesting higher arterial stiffness in this population.^39^

When arterial stiffness was measured by augmentation index (AIx), SAF was correlated with Framingham's CVD risk scores (FRS), hemoglobin, body mass index, and CVD and was inversely related to percent handgrip strength (HGS). Aortic stiffness and SAF both correlated with mortality, independent of FRS. Although all three markers, FRS, SAF, and AIx, were found to correlate with higher mortality, the extra augmentation of SAF and AIx above the risk calculated by FRS was relatively modest.^40^

A significant relationship between SAF and aortic stiffness measured by carotid–femoral PWV in subjects on chronic dialysis was found and noted to be higher in PD subjects.^41^

In children with CKD, SAF was positively associated with biochemical vascular risk markers, including matrix metalloproteinase 9, asymmetric dimethyl arginine, soluble E‐selectin, tissue inhibitor of metalloproteinase 1, symmetric dimethyl arginine, and plasminogen activator inhibitor type 1 levels.^42^

When subjects with CKD 3–5 were examined with computed tomography to estimate total coronary artery calcium score (CACS), SAF exhibited a notable relationship with vascular calcification and held significance in predicting CACS ≥400 when adjusting for age, sex, phosphate, albumin, serum calcium, C‐reactive protein, lipids, parathyroid hormone, blood pressure, and eGFR^43^ span.

A positive correlation was also seen between the SAF and CACS in PD population when SAF correlated negatively with ultrasound elastography and muscle density, where the latter correlated negatively with CACS.^44^

Vascular ultrasound performed in CKD stage 3 and nondiabetic non‐CKD controls showed a SAF score of >2.0 arbitrary units was correlated with a significantly higher risk of atheromatous plaque. When vascular age was assessed through SAF, subjects with CKD were almost 12 years older than control subjects. SAF was also negatively correlated with GFR and LDL‐cholesterol and positively correlated with age and SCORE (Systematic COronary Risk Evaluation) risk. In addition, a stepwise multivariate regression analysis showed that age and GFR independently predicted SAF.^45^

HD subjects had significantly higher SAF values and lower levels of flow‐mediated vasodilation by ultrasound than the non‐CKD controls suggesting an impaired endothelial function.^16^

When diastolic function was assessed using tissue velocity imaging on echocardiography, SAF, but not plasma autofluorescence (PAF) levels, were independently associated with diastolic dysfunction in dialysis patients. SAF was invariably correlated with age, albumin, and total protein.^46^ This may support the concept that tissue AGE explains part of the increased prevalence of diastolic dysfunction in these patients, as tissue deposition may indicate a protracted exposure to plasma levels of AGE. This ambiguous relation between plasma and tissue AGE needs further exploring.

### 2.3 Nutrition

A large study of dialysis subjects showed smoking, dialysis vintage, diabetes, serum albumin, HGS, and protein intake all to be independent predictors of elevated SAF. SAF did not correspond with dietary AGE consumption. Malnourished subjects had significantly lower dietary AGE consumption than those who were well‐nourished, but also had higher SAF.^47^ When supported by individualized nutritional guidance and assistance over 6 months, SAF levels were stable despite a rise in dietary AGE intake.^48^

A prospective observational study of 151 subjects on dialysis over 1–2 years revealed that higher baseline SAF levels and malnourishment both independently correlated with enhanced mortality.^49^

Calorie, protein, and AGE consumption hardly affected the 1‐year raise of SAF in HD subjects. However, BMI of HD subjects around 24 kg/m^2^ caused a lower 1‐year raise of SAF, indicating that AGE tissue deposition is likely related to metabolic alteration rather than mere intake.^50^

In two studies of subjects on HD, vegetarian diet negatively correlated with SAF, implying that a vegetarian diet may lessen exposure to preformed dietary and/or improve metabolism of AGE.^2^, ^21^

In almost all studies, the arm that was used to check on SAF levels and the skin color of subjects were not reported. Only two studies demonstrated that the SAF was lower in the arteriovenous fistula arm.^51^ It is unclear whether this reflects a difference in the blood supply. The value of measuring SAF in subjects with highly pigmented skin is also unclear.^52^

## 3 CONCLUSIONS

SAF was elevated in subjects on both HD and PD, and was significantly associated with cardiovascular events, cardiovascular mortality, and all‐cause mortality in CKD patients. Studies also provided a correlation between SAF and arterial stiffness, vascular damage, and subclinical atherosclerosis in these patients. SAF remained an independent predictor of graft loss and mortality as separate endpoints in kidney transplant recipients. Vegetarian diet was associated with lower levels of SAF, whereas malnutrition was correlated with higher SAF levels and mortality. The measurement of SAF may be useful in managing kidney disease. Future studies are needed to clarify the specific role of SAF in the management of CKD.

## CONFLICT OF INTEREST

The authors declare that they have no conflicts of interest to disclose.

## FINANCIAL DISCLOSURE

The authors declared that this study has received no financial support.

## Data Availability

The data that support the findings of this study are available from the corresponding author upon reasonable request.
